# Effect of different modalities of transcranial magnetic stimulation on Parkinson’s patients cognitive impairment and long-term effectiveness: a systematic review and network meta-analysis

**DOI:** 10.3389/fnins.2024.1354864

**Published:** 2024-03-01

**Authors:** Yulin Yang, Zhenyang Yan, Wanpeng Chang, Jiangtao Ding, Hongli Xu

**Affiliations:** ^1^College of Rehabilitation Medicine, Shandong University of Traditional Chinese Medicine, Jinan, China; ^2^Weifang Hospital of Traditional Chinese Medicine, Weifang, China

**Keywords:** Parkinson’s disease, transcranial magnetic stimulation, high frequency, low frequency, theta burst stimulation, cognitive impairment, network meta-analysis

## Abstract

**Objective:**

This study used network Meta-analysis to compare the effects of different transcranial magnetic stimulation (TMS) modalities on the effectiveness and long-term validity of improving cognitive function in Parkinson’s patients.

**Methods:**

Computer searches of the Cochrane Library, PubMed, Web of Science, Embass, CNKI and Wanfang Data were conducted to collect randomized controlled clinical studies on TMS to improve cognitive function in Parkinson’s patients published from the time of library construction to December 2023.

**Results:**

A total of 22 studies and 1,473 patients were included, comprising 5 interventions: high frequency repetitive transcranial magnetic stimulation (HF-rTMS), low frequency repetitive transcranial magnetic stimulation (LF-rTMS), intermittent theta burst stimulation (iTBS), sham stimulation and conventional rehabilitation therapy (CRT). Network Meta-analysis showed that the ranking results of different TMS intervention modalities in terms of MoCA scores were: HF-rTMS > LF-rTMS > iTBS > sham > CRT, the ranking results of different TMS intervention modalities in terms of MMSE scores were: HF-rTMS > LF-rTMS > sham > CRT. The effect of TMS on improving Parkinsonian cognitive function lasted for 1 month compared to the no-stimulation group.

**Conclusion:**

TMS has some long-term sustained effects on improving cognitive function in Parkinson’s patients. HF-rTMS is more effective in improving cognitive function in Parkinson’s patients.

**Systematic review registration**: https://www.crd.york.ac.uk/PROSPERO, identifier: CRD42023463958.

## Introduction

Parkinson’s disease (PD) is a degenerative disease that occurs in the elderly, with a prevalence of about 0.3%, and has become the fastest growing neurological disease in the world (Ding et al., 2022). In clinical practice, Parkinson’s patients are often accompanied by Non motor symptoms (NMS) such as depression, anxiety, and cognitive impairment. Cognitive dysfunction is the most common form of NMS, with a prevalence rate of up to 35, and 10% will develop into dementia, which is an important factor affecting the efficiency of rehabilitation and the improvement of daily life of PD patients (Hely et al., 2008; Picillo et al., 2014). The pathogenesis of cognitive impairment in PD patients is currently unclear, but it is closely related to the complex neuropathology of PD (Tansey et al., 2022). Patients with PD patients develop pathological changes such as reduction of cerebral neurons, mitochondrial dysfunction, alteration of small cerebral blood vessels, cerebral metabolism, and cortical atrophy, which can cause cortical and subcortical neurotransmitter disorders and damage to cerebral circuits, leading to cognitive dysfunction (Ashraghi et al., 2016). Currently, there is no cure for PD, and clinical treatment measures based on drugs and surgery have shortcomings such as poor efficacy, high price, and many side effects. Therefore, the search for safe and effective therapeutic measures is still a hot spot in clinical research.

Transcranial magnetic stimulation, as a non-invasive and safe peripheral intervention, is able to stimulate nerve cells in the brain based on electromagnetic induction, creating painless electrical currents to increase the excitability of cortical nerve cells in patients with PD patients, or to improve cognitive function by repairing abnormal neuroplasticity (Elahi et al., 2009; Lefaucheur et al., 2020). Long-term TMS stimulation has a cumulative effect and has a long-term therapeutic effect in PD patients (Ridding and Rothwell, 2007). TMS can be categorized into various modes according to the form of pulses, and the commonly used clinical modes are repetitive transcranial magnetic stimulation (rTMS) and theta burst stimulation (TBS). For rTMS, the choice of stimulation site, stimulation frequency, stimulation intensity, and treatment duration are all important factors affecting its therapeutic effect. Generally, magnetic stimulation with a frequency of ≤1.0 Hz is called low-frequency stimulation, which mainly reduces the excitability of the cerebral cortex, while high-frequency stimulation with a frequency of >1.0 Hz can increase the excitability of the cerebral cortex (Fitzgerald et al., 2006; Rothkegel et al., 2010). Theta burst stimulation can be divided into continuous theta burst stimulation (cTBS) and iTBS stimulation modes, which are also capable of exerting inhibitory or excitatory effects on the cortex, respectively (Pascual-Leone et al., 2000; Huang et al., 2005). However, due to a lack of understanding of the mechanisms underlying the sustained repair of stimulus-induced cortical excitability and inter-subject heterogeneity, the duration of the therapeutic effect of TMS applied to patients’ cerebral cortex is unclear and there is no consensus on the overall efficacy of the different modalities of TMS for the treatment of cognitive dysfunction in PD patients.

Chen et al. (2014) study showed that high-frequency rTMS was more effective than low-frequency rTMS in improving motor and depressive symptoms in PD patients, and Shirota et al. (2013) study showed that low-frequency was more effective. Trung et al. (2019) performed rTMS stimulation on PD patients with mild cognitive impairment and showed that the effect of cognitive improvement lasted for 1 month. Yang (2023) followed up Parkinson’s patients after rTMS stimulation and found that the effect of cognitive improvement lasted for 2 months. In contrast, stimulation parameters were not found to have any significant effect on cognitive function in a meta-analysis conducted by Goodwill et al. (2017). Thus, the effect of TMS on cognitive function in PD patients remains controversial, and the optimal parameters and stimulation modes remain unclear. To address these issues, the aim of this study was to provide an objective and comprehensive analysis to determine the duration of improvement in cognitive function and the optimal modes of intervention of TMS therapy for the treatment of patients with PD patients. Since there were no reports of cTBS in the retrieved studies, this paper mainly included the studies related to HF-rTMS, LF-rTMS, and iTBS to explore the application of TMS in the clinic for the presence of in this paper, we included studies on HF-rTMS, LF-rTMS, and iTBS to explore the optimal intervention mode of TMS in PD patients with cognitive impairment, with the aim of providing an evidence-based basis for the better application of TMS in PD patients with cognitive impairment.

## Materials and methods

### Search strategy

The Cochrane Library, PubMed, Web of Science, Embass, CNKI, Wanfang Data databases were searched by two independent researchers using subject terms combined with free words from the time of their creation to December 2023. In addition, supplementary manual searches of references and grey literature included in the studies were conducted to ensure the comprehensiveness of the search. The search terms included: Parkinson’s diseases, transcranial magnetic stimulation, high frequency, low frequency, theta burst stimulation, cognitive impairment, randomized controlled studies.

### Inclusion criteria

A randomized controlled trial of the application of TMS in Parkinsonian patients. The study subjects were patients with PD patients with cognitive dysfunction, all of them were > 18 years old, and they were conscious and had stable vital signs. The control group was CRT, CRT mainly consists of conventional drug therapy, occupational therapy and physical therapy commonly used in clinical practice. The experimental group was treated with TMS on the basis of CRT. TMS modes included HF-rTMS, LF-rTMS, and iTBS. The primary outcomes were the Montreal Cognitive Assessment (MoCA) and the Mini-Mental State Examination (MMSE), and the secondary outcomes were the Unified Parkinson Disease Rating Scale part I and II (UPDRS I, II).

### Exclusion criteria

Non-English and Chinese literature, duplicate publications, studies with non- randomized controlled trials (RCTs) designs, no baseline conditions, incomplete or unextractable raw data and fruitless attempts to contact the authors, conference papers, master’s and doctoral dissertations, and literature without specified outcome indicators.

### Data extraction

The retrieved literature was imported into EndNote software and duplicates were excluded. 2 investigators independently screened and extracted data according to inclusion and exclusion criteria and cross-checked the literature, consulting a third investigator in case of disagreement. The data extraction included basic information, baseline conditions, interventions, and outcome indicators. All outcomes were included in the pre-treatment and post-treatment differences.

### Quality assessment

The quality of the included literature was assessed by two investigators using the Cochrane Handbook of Systematic Reviews 5.1.0. The assessment included (1) Selective bias: assessed by the appropriateness of the random sequence generation method and the perfection of the allocation scheme concealment; (2) Implementation bias: blinding of subjects or researchers and outcome assessors, assessed by the perfection of the blinding method; (3) Follow-up bias: assessed by whether the outcome data were complete and whether missing data were handled appropriately; (4) Reporting bias: assessed by whether the results were selectively reported; (5) Other bias. The risk of bias was evaluated as high, low, or unclear.

### Statistical analysis

The STATA 17.0 software was used to conduct the reticulated Meta-analysis, and the included outcome indicators were continuous variables, and the effect size was expressed by mean difference (MD), and 95% confidence interval (CI) was calculated as the effect size, while the *I*^2^ statistic was combined to quantitatively determine the size of heterogeneity. When *p* ≥ 0.05, *I^2^*≦50%, it was considered that there was no heterogeneity among studies, and when *p* < 0.05, *I*^2^ > 50%, it was considered that there was significant heterogeneity among studies. For the heterogeneous results, the sources of heterogeneity could be explored by subgroup analysis, sensitivity analysis or by conducting qualitative descriptions. Stata was used to plot the network evidence of each outcome indicator, each dot represented different interventions, the dots represented number of cases for that intervention, the line connected between the two dots is the evidence for the existence of direct comparisons between the two interventions, and the thickness of the solid line is the amount of evidence for direct comparisons. Inconsistency tests were performed using nodal analysis, and if *p* > 0.05, the difference between direct and indirect comparisons was not statistically significant and the two results were consistent, and the analysis was performed using the consistency model, and vice versa. This study used Stata to detect the inconsistency factor and 95% CI in the closed loop, and the inconsistency factor (IF) was small when the 95% CI contained 0, suggesting that the evidence for direct versus indirect comparisons was very consistent. The ranking status of each outcome indicator was expressed using a cumulative probability plot, with a larger area under the curve of the cumulative probability plot representing better efficacy.

## Results

### Study selection

**Figure 1:**
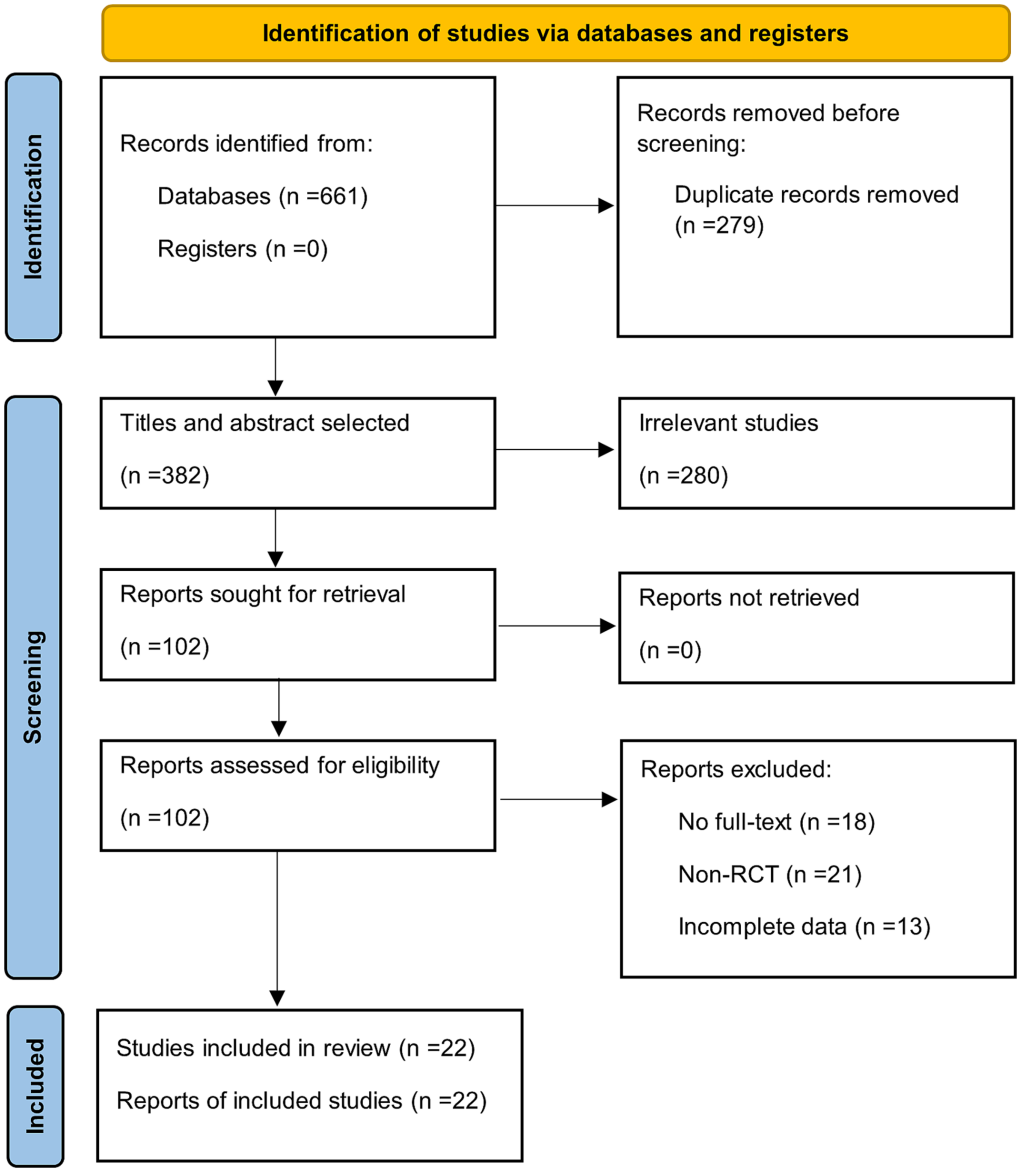
PRISMA flow-chart showing selection of articles for network meta-analysis.

Six databases were searched according to the search formula, and a total of 661 papers were obtained from the preliminary search, including 64 papers from Cochrane, 150 papers from PubMed, 229 papers from Web of Science, 107 papers from Embass, 50 papers from CNKI and 61 papers from Wanfang Data. Twenty-two papers (Su et al., 2012; Tang et al., 2015; Yu et al., 2017; Dong and Yang, 2018; Zhu et al., 2018; Li et al., 2019; Wu et al., 2019; Chi, 2020; Guo, 2020; Jiang L. et al., 2020; Khedr et al., 2020; Lang et al., 2020; Li, 2020; Liu and Lu, 2020; Zhang et al., 2020; Zhuang et al., 2020; He et al., 2021; Liao et al., 2021; Cheng et al., 2022; Gao et al., 2022; Huang et al., 2023; Yang, 2023) were finally included after eliminating duplicates and reading through the titles, abstracts, and full text and quality assessment. The detailed screening process and the reasons for exclusion are shown in Figure. The detailed screening process and reasons for exclusion are shown in Figure 1, and the basic characteristics of the included literature are shown in Tables 1, 2.

**Table 1:** Characteristics of the included studies.

Studies	Country	Age (x ± s, year)		Duration (x ± s, year)		Sample (*n*)	
		T	C	T	C	T/C	
Dong and Yang (2018)	China	65.83 ± 11.57	65.83 ± 11.57	7.30 ± 2.40	7.30 ± 2.40	30	30
Gao et al. (2022)	China	65.30 ± 6.24	65.25 ± 6.32	4.10 ± 1.12	4.05 ± 1.20	40	49
Huang et al. (2023)	China	63.37 ± 5.56	62.74 ± 5.48	4.12 ± 1.46	3.94 ± 1.31	51	51
Khedr et al. (2020)	Egypt	65.56 ± 8.73	59.33 ± 10.27	5.89 ± 5.37	5.50 ± 3.85	18	15
Li et al. (2019)	China	54.51 ± 3.49	54.54 ± 3.51	3.13 ± 0.55	3.12 ± 0.53	41	41
Liao et al. (2021)	China	59.03 ± 6.84	60.43 ± 6.94	–	–	30	30
Liu and Lu (2020)	China	–	–	–	–	27	27
Wu et al. (2019)	China	59.60 ± 6.10	60.50 ± 5.80	5.80 ± 1.60	5.50 ± 1.40	50	50
Yang (2023)	China	63.26 ± 7.39	63.61 ± 7.85	6.77 ± 2.66	6.97 ± 2.78	56	56
Yu et al. (2017)	China	60.32 ± 9.63	60.16 ± 10.14	2.62 ± 0.86	2.33 ± 0.74	31	30
Zhu et al. (2018)	China	62.37 ± 5.18	63.20 ± 5.34	3.31 ± 0.95	3.24 ± 1.01	57	57
Zhang et al. (2020)	China	59.74 ± 4.84	59.42 ± 5.02	3.45 ± 0.77	3.62 ± 0.81	37	37
Chi (2020)	China	65.21 ± 4.39	65.19 ± 4.40	6.12 ± 1.71	6.21 ± 1.74	50	50
Guo (2020)	China	65.91 ± 3.42 66.28 ± 3.55	66.57 ± 3.39	6.48 ± 2.08 6.15 ± 1.97	6.64 ± 2.11	38	38
Jiang L. et al. (2020)	China	67.49 ± 7.83	65.49 ± 7.43	–	–	18	18
Li (2020)	China	61.90 ± 5.30	61.60 ± 5.40	5.20 ± 1.30	5.30 ± 1.20	43	43
Su et al. (2012)	China	57.30 ± 5.90	59.10 ± 4.70	6.50 ± 1.40	6.90 ± 1.70	29	29
Tang et al. (2015)	China	68.50 ± 11.20	68.50 ± 11.20	6.80 ± 2.50	6.80 ± 2.50	25	25
Zhuang et al. (2020)	China	60.58 ± 9.21	61.57 ± 13.25	8.53 ± 2.07	7.71 ± 2.27	19	14
Cheng et al. (2022)	China	71.60 ± 5.10	73.90 ± 6.90	–	–	11	16
He et al. (2021)	China	70.00 ± 6.30	74.80 ± 6.90	2.70 ± 1.50	2.50 ± 1.10	20	15
Lang et al. (2020)	Canada	68.43 ± 8.40	68.76 ± 8.30	5.95 ± 4.80	4.80 ± 4.00	20	20

T, experimental group; C, control group.

**Table 2:** Characteristics of the included studies.

**Studies**	**Intervention measures**						**Comparison measure**	**Outcomes**
	**Location**	**Frequency**	**Intensity**	**Pulse number**	**Treatment cycle**	**Coil Type**		
Dong and Yang (2018)	DDLPFC	5	80%RMT	600	4 weeks	8-shaped	Sham stimulation	①②
Gao et al. (2022)	LDLPFC	10	80%RMT	500	4 weeks	8-shaped	CRT	①②
Huang et al. (2023)	LDLPFC	8	85%RMT	–	4 weeks	–	Sham stimulation	①②
Khedr et al. (2020)	M1	20	90%RMT	–	2 weeks	8-shaped	CRT	①②③④
Li et al. (2019)	DDLPFC	5	80%RMT	600	4 weeks	-	Sham stimulation	①②⑤⑥
Liao et al. (2021)	LDLPFC	10	90%RMT	500	4 weeks	8-shaped	CRT	①
Liu and Lu (2020)	DDLPFC	5	80%RMT	600	4 weeks	–	Sham stimulation	①
Wu et al. (2019)	LDLPFC	5	–	1,600	4 weeks	Circle	CRT	②⑤⑥
Yang (2023)	RDLPFC	25	80%RMT	1,350	20 times	8-shaped	CRT	①②③④
Yu et al. (2017)	M1	5	100% RMT	1,600	4 weeks	8-shaped	CRT	②④
Zhu et al. (2018)	Extremity movement area	5	110%RMT	1,600	4 weeks	–	Sham stimulation	①②
Zhang et al. (2020)	LDLPFC	10	2.2Tesla	800	12 weeks	8-shaped	CRT	①
Chi (2020)	LDLPFC	0.5	100%RMT	–	4 weeks	–	Sham stimulation	①②
Guo (2020)	M1	5/1	100% RMT	1,600	10 days	–	CRT	②④
Jiang L. et al. (2020)	–	1	–	1,800	10 days	Circle	Sham stimulation	④
Li (2020)	RDLPFC	1	60%RMT	1,200	2 weeks	8-shaped	Sham stimulation	②⑤⑥
Su et al. (2012)	DDLPFC	0.5	80%RMT	–	20 days	–	CRT	②④
Tang et al. (2015)	M1	1	120%RMT	–	4 weeks	8-shaped	Sham stimulation	②⑤⑥
Zhuang et al. (2020)	LDLPFC	1	110%RMT	1,200	10 days	8-shaped	CRT	①③
Cheng et al. (2022)	LDLPFC	iTBS	90%RMT	600	2 weeks	8-shaped	CRT	①
He et al. (2021)	LDLPFC	iTBS	100%RMT	–	2 weeks	8-shaped	CRT	①③
Lang et al. (2020)	LDLPFC	iTBS	80%RMT	600	4 weeks	8-shaped	CRT	①

CRT, conventional rehabilitation therapy; RMT, resting motor threshold; DLPFC, dorsolateral prefrontal cortex; DDLPFC, Bilateral DLPFC; LDLPFC, left DLPFC; RDLPFC, right DLPFC; M1, primary motor cortex; ① MoCA, ② MMSE, ③ MoCA follow-up, ④ MMSE follow-up, ⑤ UPDRS-I, ⑥ UPDRS-II.

### Quality assessment

**Figure 2:**
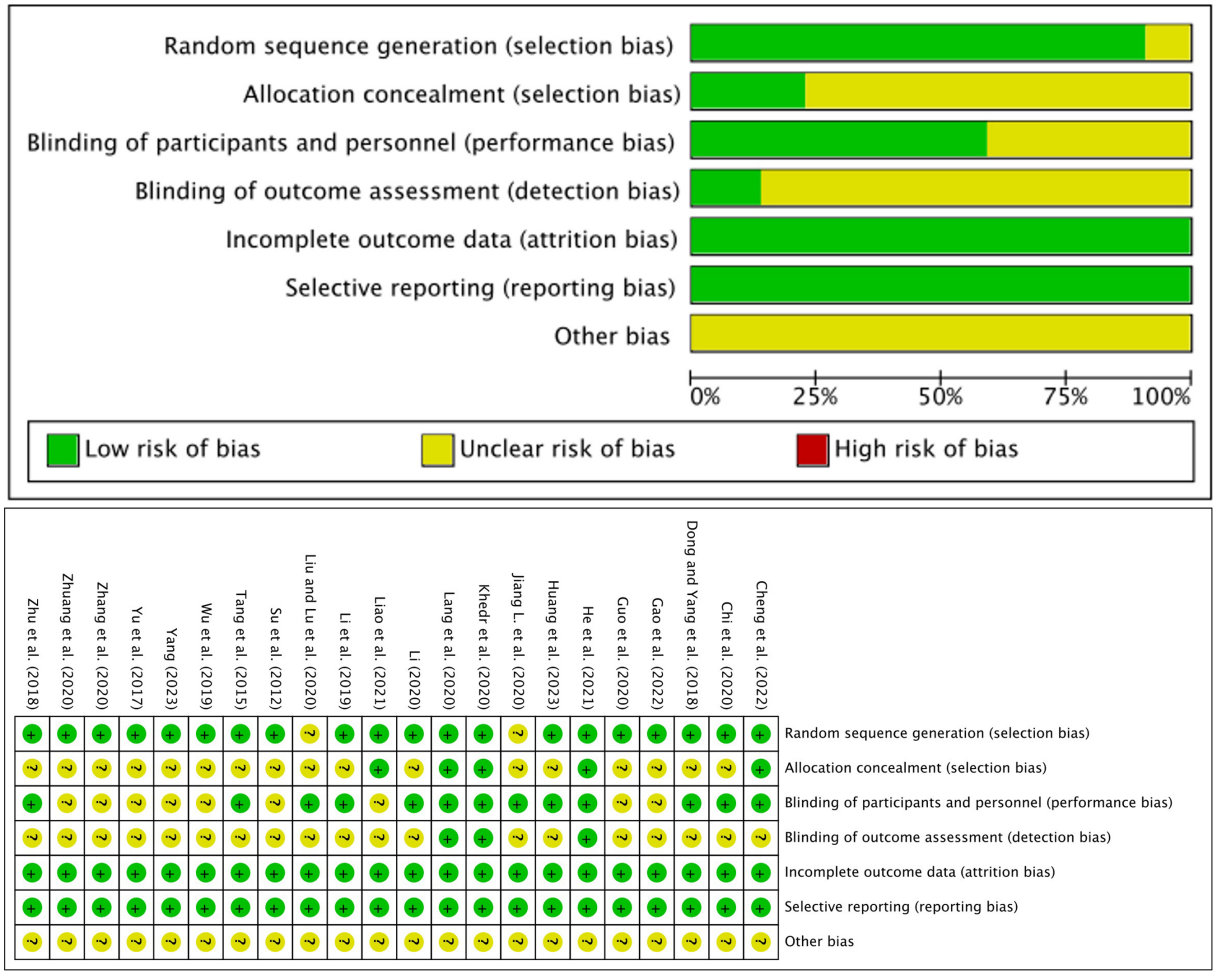
Risk assessment of bias.

The Cochrane Handbook of Systematic Reviews 5.1.0 was used to assess the quality of the included literature. Of the 22 included papers, 22 referred to “randomized groups” and 20 (Tang et al., 2015; Yu et al., 2017; Dong and Yang, 2018; Zhu et al., 2018; Li et al., 2019; Wu et al., 2019; Chi, 2020; Guo, 2020; Khedr et al., 2020; Lang et al., 2020; Li, 2020; Liu and Lu, 2020; Zhang et al., 2020; Zhuang et al., 2020; He et al., 2021; Liao et al., 2021; Cheng et al., 2022; Gao et al., 2022; Huang et al., 2023; Yang, 2023) described the specific randomization method. Five papers (Khedr et al., 2020; Lang et al., 2020; He et al., 2021; Liao et al., 2021; Cheng et al., 2022) had an “opaque envelope” allocation concealment. Thirteen papers (Tang et al., 2015; Dong and Yang, 2018; Zhu et al., 2018; Li et al., 2019; Chi, 2020; Jiang L. et al., 2020; Khedr et al., 2020; Lang et al., 2020; Li, 2020; Liu and Lu, 2020; He et al., 2021; Cheng et al., 2022; Huang et al., 2023) were blinded to study subjects or interventionists. Three papers (Khedr et al., 2020; Lang et al., 2020; He et al., 2021) were blinded to outcome measures. Twenty-two of the literature had complete outcome data, no selective reporting of outcomes, and other risks of bias were unclear, as detailed in Figure 2.

### Network meta-analysis

#### Evidence network

**Figure 3:**
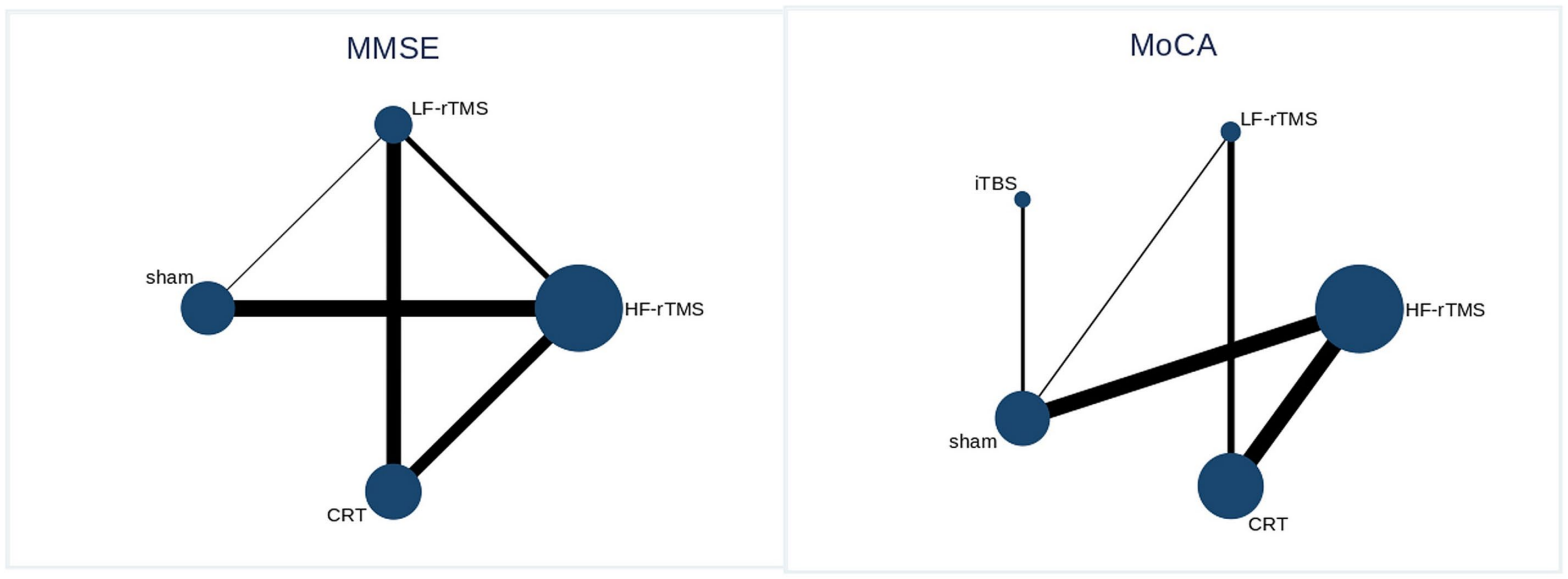
NMA of MoCA and MMSE scores at different TMS.

Fourteen studies (Dong and Yang, 2018; Zhu et al., 2018; Li et al., 2019; Chi, 2020; Khedr et al., 2020; Lang et al., 2020; Liu and Lu, 2020; Zhuang et al., 2020; He et al., 2021; Liao et al., 2021; Cheng et al., 2022; Gao et al., 2022; Huang et al., 2023; Yang, 2023) involved MoCA scores and included five rehabilitation treatments, 14 studies (Su et al., 2012; Tang et al., 2015; Yu et al., 2017; Dong and Yang, 2018; Zhu et al., 2018; Li et al., 2019; Wu et al., 2019; Chi, 2020; Guo, 2020; Khedr et al., 2020; Li, 2020; Gao et al., 2022; Huang et al., 2023; Yang, 2023) involved MMSE scores and included four rehabilitation treatments. Each dot represents a different intervention, with larger dots indicating more cases of that intervention and vice versa. The line connecting the two dots is evidence of a direct comparison between the two interventions, and vice versa. The thicker the solid line, the more evidence of direct comparison and vice versa (Figure 3).

#### Inconsistency test

**Figure 4:**
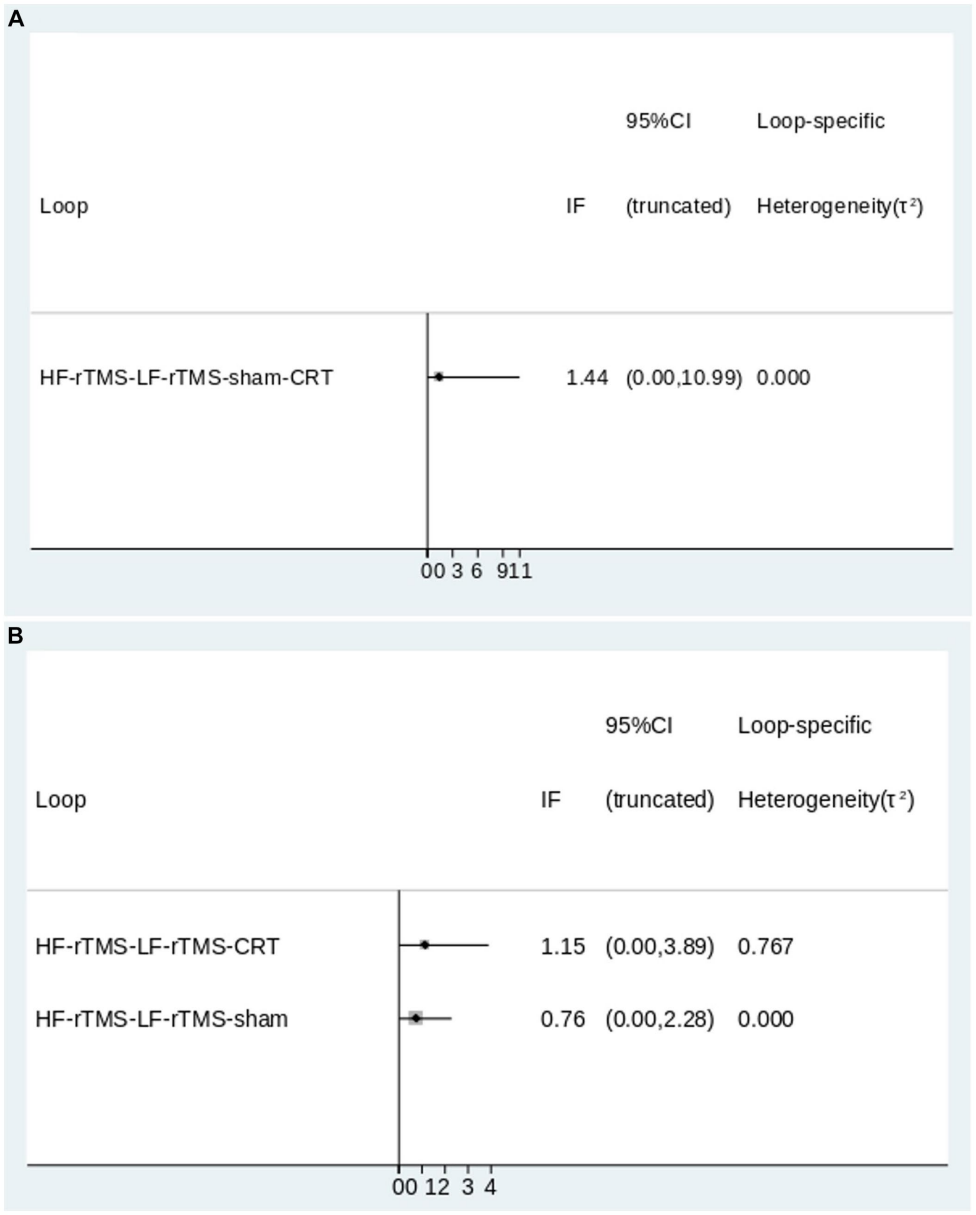
The inconsistency plot for direct and indirect comparisons (**A**: MoCA; **B**: MMSE).

Inconsistency tests were performed on the closed loops formed by the MoCA and MMSE outcome indicators, and when the 95% CI contained 0, the inconsistency factor (IF) was small, indicating little heterogeneity. The results showed an inconsistency factor of 1.44 for MoCA and 1.15 and 0.76 for MMSE, with the lower limit of the 95% CI containing 0, suggesting that the consistency of the closed loop of the outcome metrics was relatively good and the network meta-analysis was reliable (Figure 4).

### The results of network meta-analysis

#### MoCA

Direct and indirect comparisons of the five rehabilitation treatment measures showed significant differences in one of the groups. HF-rTMS (MD = –4.54, 95% CI = –6.61 to −2.48) significantly improved MoCA scores compared to conventional rehabilitation (*p* < 0.05), with no significant difference between the remaining groups (*p* > 0.05) (Table 3).

**Table 3:** Network meta-analysis of MoCA (MD [95% CI]).

MD (95%CI)				
HF-rTMS				
−0.68(−5.21,3.85)	LF-rTMS			
−1.16(−4.63,2.31)	−0.48(−5.05,4.08)	iTBS		
−2.33(−5.03,0.37)	−1.65(−5.59,2.29)	−1.17(−3.50,1.17)	Sham	
−4.54(−6.61,-2.48)*	−3.86(−8.68,0.96)	−3.38(−7.37,0.61)	−2.21(−5.57,1.14)	CRT

*The values between the 2 comparisons are statistically significant.HF-rTMS, high frequency repetitive transcranial magnetic stimulation; LF-rTMS, low frequency repetitive transcranial magnetic stimulation; iTBS, intermittent theta burst stimulation, sham: sham stimulation; CRT, conventional rehabilitation therapy.

#### MMSE

Direct and indirect comparisons of the four rehabilitation treatment measures showed significant differences in four of the groups. HF-rTMS (MD = –3.63, 95% CI = –4.38 to −2.87) and LF-rTMS (MD = –2.89, 95% CI = –3.76 to −2.03) significantly improved MMSE scores compared to conventional rehabilitation (*p* < 0.05), HF-rTMS (MD = –3.13, 95% CI = –3.94 to −2.33) and LF-rTMS (MD = –2.40, 95% CI = –3.44 to −1.35) significantly improved MMSE scores compared to the sham stimulation group, with no significant difference between the remaining groups (*p* > 0.05) (Table 4).

**Table 4:** Network meta-analysis of MMSE (MD [95% CI]).

MD (95%CI)			
HF-rTMS			
−0.73(−1.69,0.22)	LF-rTMS		
−3.13(−3.94,-2.33)*	−2.40(−3.44,–1.35)*	sham	
−3.63(−4.38,-2.87)*	−2.89(−3.76,−2.03)*	−0.49(−1.51,0.52)	CRT

*The values between the 2 comparisons are statistically significant; HF-rTMS, high frequency repetitive transcranial magnetic stimulation; LF-rTMS, low frequency repetitive transcranial magnetic stimulation; sham, sham stimulation; CRT, conventional rehabilitation therapy.

#### Surface under the cumulative ranking

**Figure 5:**
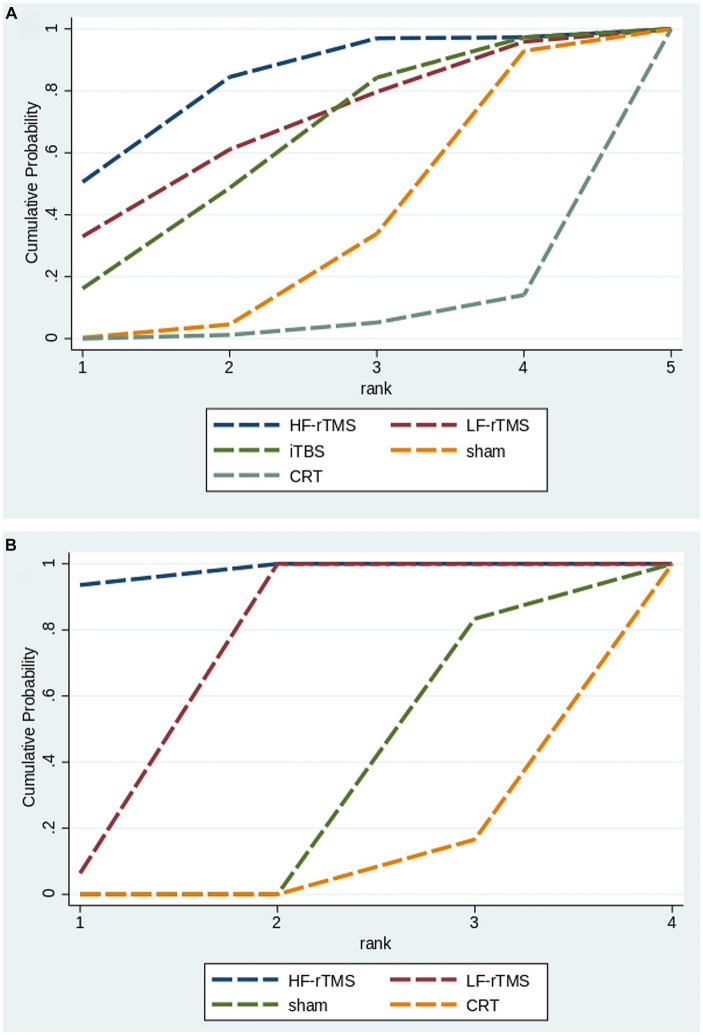
Surface under the cumulative ranking curve of all included TMS (**A**: MoCA; **B**: MMSE).

The cumulative probability ranking results showed that high-frequency repetitive transcranial magnet may be the best intervention modality for improving MoCA scores (HF-rTMS [50.6%] > LF-rTMS [33.0%] > iTBS [16.2%] > sham stimulation [0.3%] > CRT [0.0%]), and high-frequency repetitive transcranial magnet may be the best intervention modality for improving MMSE scores (HF-rTMS [93.6%] > LF-rTMS [6.4%] > sham stimulation [0.0%] > CRT [0.0%]) (Figure 5).

#### Publication bias

**Figure 6:**
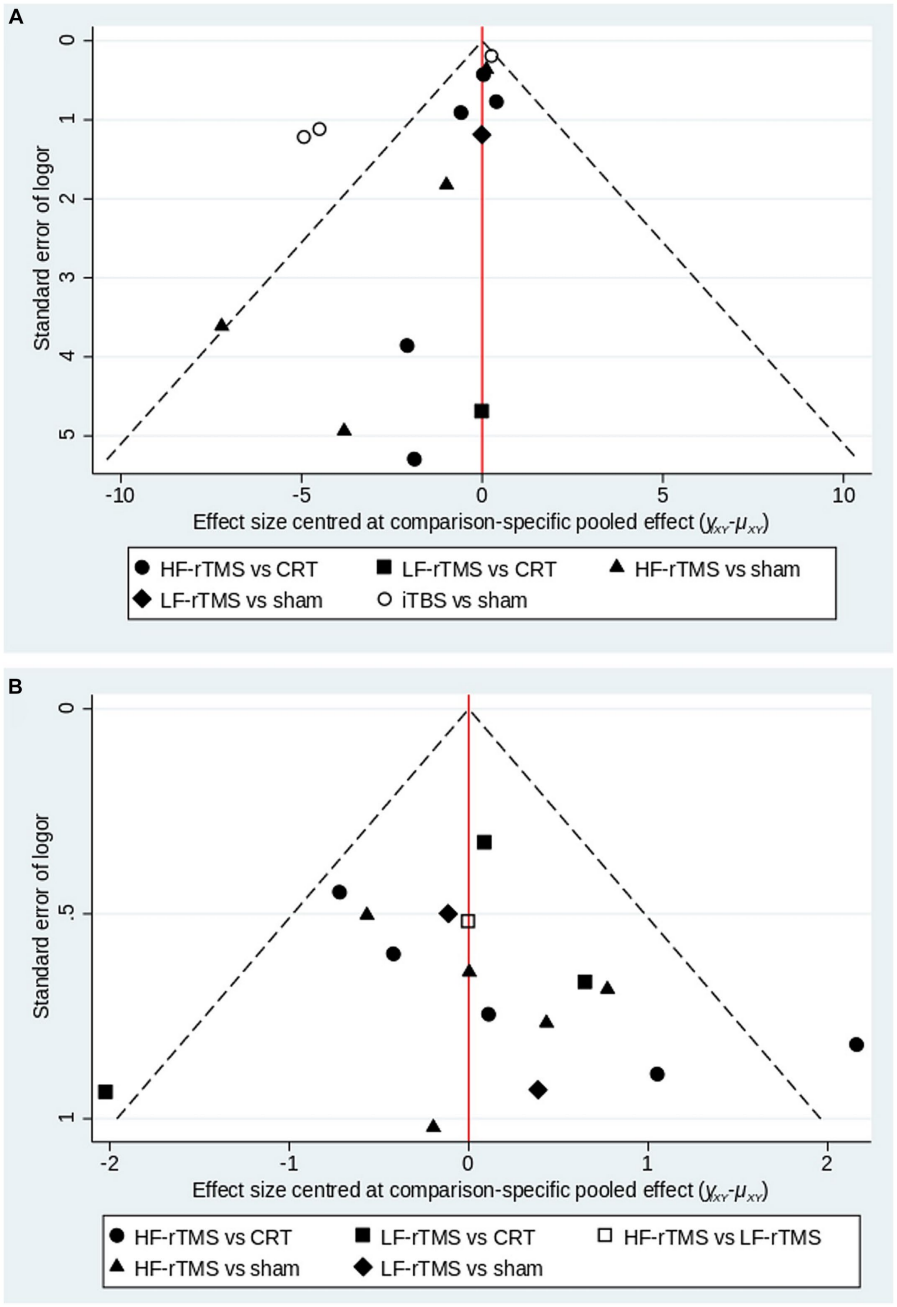
A funnel plot to confirm the risk of publications bias for included literatures (**A**: MoCA; **B**: MMSE).

This article examined publication bias in the MoCA and MMSE outcome metrics, where different shapes in the funnel plot represent direct comparisons between two different stimulus modalities, and the number of shapes represents the number of studies in that stimulus modality. The results show that the funnel plots are roughly symmetrical, indicating a low likelihood of publication bias in the articles, and that two studies in each of the MoCA and MMSE are distributed outside the 95% CI of the funnel plots, suggesting that there may be a small sample effect (Figure 6).

### Follow up

#### MoCA

**Figure 7:**
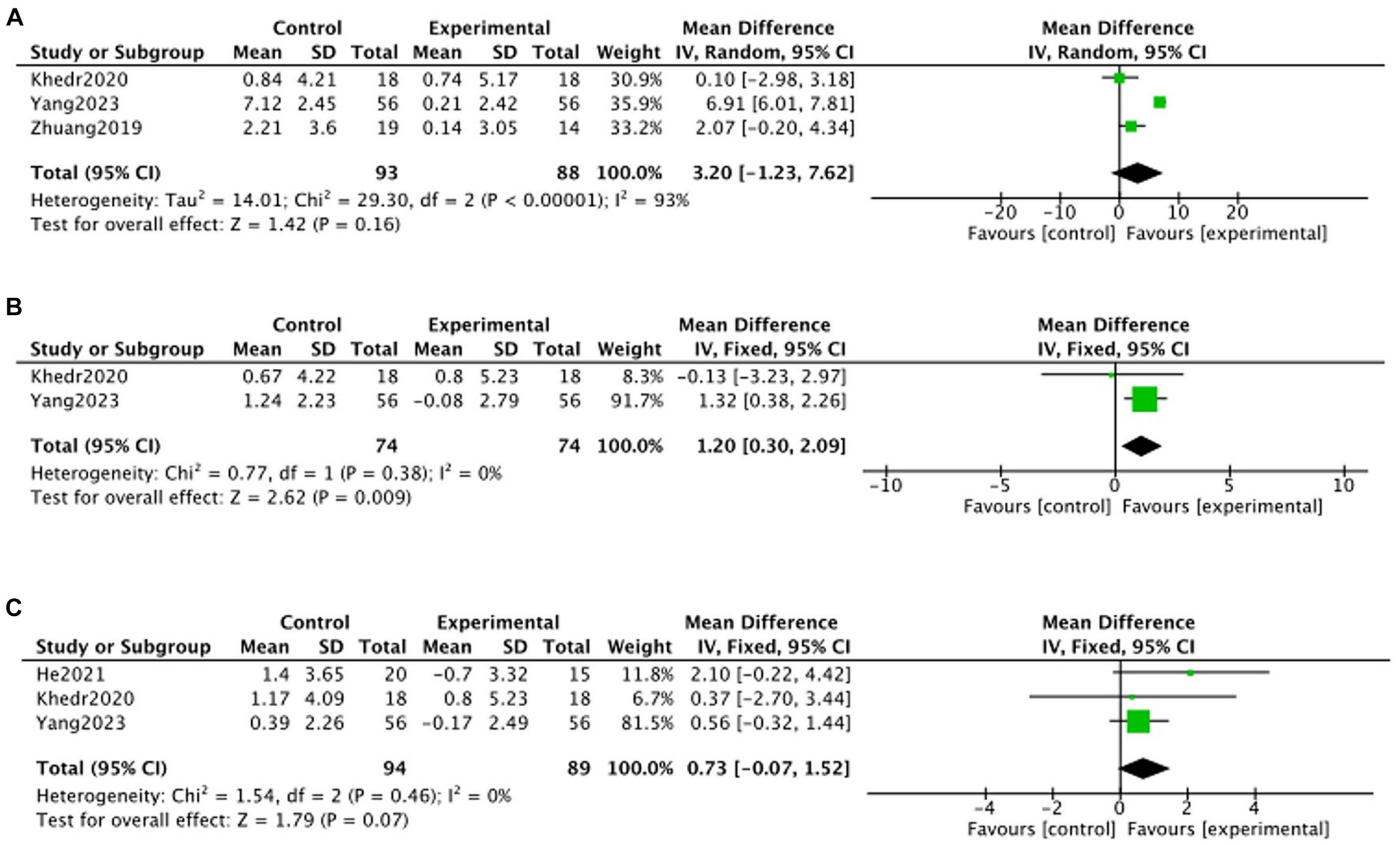
Forest plot of MoCA changes in TMS and no stimulation groups (**A**: 1 month; **B**: 2 months; **C**: 3 months).

Due to the limited number of included articles, this study was not specifically categorized by stimulation modality and only reported the overall effect of TMS treatment on MoCA changes. Three articles (Khedr et al., 2020; Zhuang et al., 2020; Yang, 2023) reported MoCA c changes at 1 month post-treatment, two articles (Khedr et al., 2020; Yang, 2023) reported MoCA changes at 2 months post-treatment, and three articles (Khedr et al., 2020; He et al., 2021; Yang, 2023) reported MoCA changes at 3 months post-treatment. Meta-analysis showed no statistically significant difference in MoCA in the TMS group compared to the no-stimulation group at months 1, 3 of follow-up (month 1: MD = 3.20, 95% CI –1.23 to 7.62, *p* > 0.05; month 3: MD = 0.73, 95% CI –0.07 to 1.52, *p* > 0.05). At month 2 of follow-up, there was a statistically significant increase in MoCA in the TMS group compared to the no-stimulation group (month 2: MD = 1.20, 95% CI 0.30–2.09, *p* < 0.05) (Figure 7).

#### MMSE

**Figure 8:**
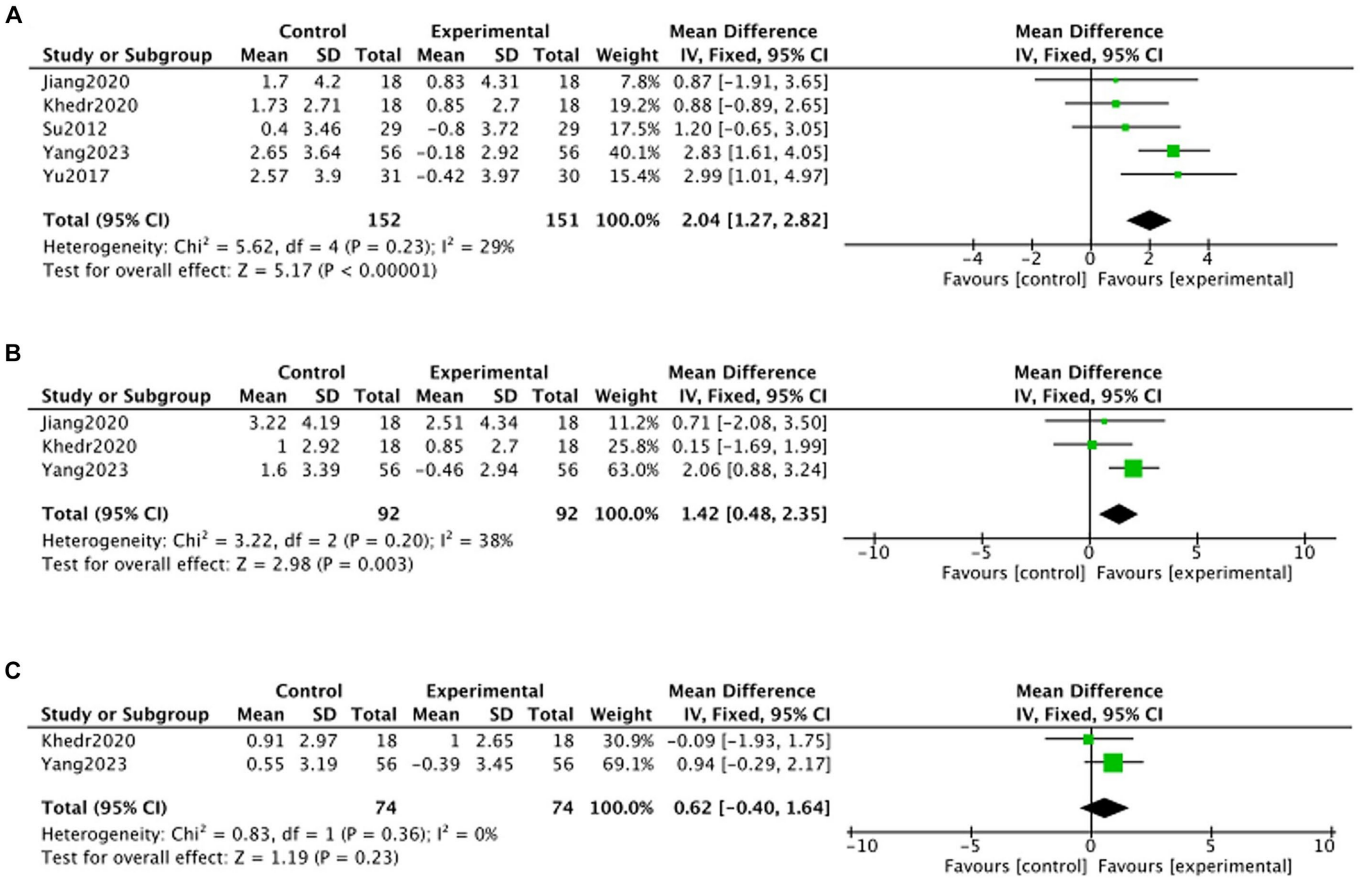
Forest plot of MMSE changes in TMS and no stimulation groups (**A**: 1 month; **B**: 2 months; **C**: 3 months).

Due to limited inclusion of articles, this study was not specifically categorized by stimulation modality and only reported on the overall effect of TMS treatment on the change in MMSE. Five articles (Su et al., 2012; Yu et al., 2017; Jiang L. et al., 2020; Jiang Y. et al., 2020; Khedr et al., 2020; Yang, 2023) reported on the change in MMSE at 1 month post-treatment, three articles (Jiang L. et al., 2020; Khedr et al., 2020; Yang, 2023) reported on the change in MMSE at 2 months post-treatment, and two articles (Khedr et al., 2020; Yang, 2023) reported on the change in MMSE at 3 months post-treatment. Meta-analysis showed that, at the 3rd month of follow-up, there was no statistically significant difference in MMSE in the TMS group compared to the no-stimulation group (month 3: MD = 0.62, 95% CI –0.40 to 1.64, p > 0.05). month, there was no statistically significant MMSE in the TMS group compared to the no-stimulation group (month 3: MD = 0.62, 95%CI –0.40 to 1.64, *p* > 0.05). At months 1 and 2 of follow-up, there was a statistically significant increase in MMSE in the TMS group compared to the no-stimulation group (month 1: MD = 2.04, 95%CI 1.27–2.82, *p* < 0.05; month 2: MD = 1.42, 95%CI 0.48–2.35, *p* < 0.05) (Figure 8).

#### Adverse reaction

Seven studies (Tang et al., 2015; Chi, 2020; Zhang et al., 2020; Liao et al., 2021; Gao et al., 2022; Huang et al., 2023; Yang, 2023) reported that a few patients experienced transient dizziness, pain, and pins and needles, and sneezing during treatment. The patients could be relieved after rest and did not affect the treatment. No adverse effects were reported in other studies.

#### Subgroup analysis of MoCA

The MoCA scale contains ratings of seven cognitive functions: visuospatial, naming, attention, language, abstraction, delayed recall and orientation. Our current subgroup analysis of the MoCA subscales from four studies (Zhang et al., 2020; He et al., 2021; Liao et al., 2021; Cheng et al., 2022) involving 2 intervention modalities is presented in Table 5.

**Table 5:** Subgroup analysis of MoCA.

Subgroup analysis		SMD (95% CI)	*P*	*I^2^*(%)
HF-rTMS	Visuospatial	0.39 [0.26, 0.53]	<0.00001	27%
	Naming	0.14 [−0.02, 0.30]	0.10	80%
	Attention	0.76 [−0.52, 2.04]	0.25	97%
	Language	0.28 [0.13, 0.42]	0.0002	36%
	Abstraction	0.18 [0.03, 0.34]	0.02	0%
	Delayed recall	0.28 [0.14, 0.42]	0.0001	0%
	Orientation	0.83 [−0.64, 2.30]	0.27	98%
iTBS	Visuospatial	0.41 [−0.06, 0.89]	0.09	0%
	Naming	0.10 [−0.26, 0.47]	0.57	68%
	Attention	0.34 [−0.01, 0.68]	0.06	0%
	Language	0.76 [0.49, 1.03]	< 0.00001	-
	Abstraction	0.04 [−0.24, 0.31]	0.79	0%
	Delayed recall	1.20 [0.47, 1.94]	0.001	0%
	Orientation	0.04 [−0.50, 0.58]	0.88	0%

SMD, standard mean difference; HF-rTMS, high frequency repetitive transcranial magnetic stimulation; iTBS, intermittent theta burst.

#### Subgroup analysis of second outcomes

Subgroup analyses of secondary outcome indicators showed that HF-rTMS had significant differences in improving both UPDRS I and UPDRS II compared to conventional rehabilitation. Among them, LF-rTMS was more effective in improving UPDRS I, and HF-rTMS was more effective in improving UPDRS II (Table 6).

**Table 6:** Subgroup analysis of second outcomes.

Outcome indicator	Intervention	Comparison number	SMD (95%CI)	*P*	*I*^2^(%)
UPDRS I	HF-rTMS VS. CRT	2	−1.23(−2.86 to 0.41)	0.14	93%
	LF-rTMS VS. CRT	2	−0.81(−1.12 to- 0.49)	<0.00001	0%
	Total	4	−1.00(−1.62 to −0.39)	0.001	80%
UPDRS II	HF-rTMS VS. CRT	2	−3.37(−4.14 to −2.60)	<0.00001	19%
	LF-rTMS VS. CRT	2	−2.24(−4.66 to 0.18)	0.07	92%
	Total	4	−2.86(−4.50 to −1.21)	0.0007	93%

SMD, standard mean difference; HF-rTMS, high frequency repetitive transcranial magnetic stimulation; LF-rTMS, low frequency repetitive transcranial magnetic stimulation; CRT, conventional rehabilitation therapy.

#### Subgroup analysis of other influencing factors

In order to explore other factors that may affect the effect of TMS, we performed subgroup analyses by stimulation site, stimulation intensity, and number of stimulation pulses in MoCA and MMSE. Because the results obtained from meta-analyses that included only a single study were highly controversial, the single data were excluded from the present study before combining with the confidence intervals to make a comprehensive judgment. The results showed that 100-intensity TMS stimulation of DDLPFC with >600 pulses demonstrated superior results in MoCA, and 100-intensity TMS stimulation of DDLPFC with ≤600 pulses demonstrated superior results in MMSE, so in summary, 100-intensity TMS stimulation of DDLPFC may have better results, but this conclusion needs to be treated with caution due to the limited number of included studies, the results are shown in Table 7.

**Table 7:** Subgroup analysis of other influencing factors.

Subgroup analysis		Studies	SMD (95% CI)	*P*	*I^2^*(%)
MoCA					
Location	LDLPFC	8	1.54 [−0.37, 3.45]	0.11	91%
	RDLPFC	1	8.26 [1.17, 15.35]	0.02	–
	DDLPFC	3	5.73 [2.67, 8.79]	0.0002	80%
	M1	1	2.04 [−1.54, 5.62]	0.26	–
Intensity (RMT)	80	6	4.20 [−1.01, 9.41]	0.11	98%
	90	3	1.80 [0.12, 3.49]	0.04	51%
	100	2	3.05 [0.92, 5.18]	0.005	0%
Pulse number	≦600	7	2.97 [−0.18, 6.12]	0.06	98%
	>600	3	4.01 [1.22, 6.80]	0.005	73%
MMSE					
Location	LDLPFC	4	3.26 [2.77, 3.75]	< 0.00001	0%-
	RDLPFC	2	3.30 [2.37, 4.24]	< 0.00001	57%
	DDLPFC	2	3.56 [1.67, 5.44]	0.0002	75%
	M1	4	3.35 [2.16, 4.53]	< 0.00001	55%
Intensity (RMT)	80	5	3.30 [2.27, 4.34]	< 0.00001	59%
	90	1	4.25 [2.50, 6.00]	< 0.00001	-
	100	3	3.58 [3.06, 4.10]	< 0.00001	0%
Pulse number	≦600	3	3.93 [2.68, 5.19]	< 0.00001	56%
	>600	6	3.16 [2.67, 3.65]	< 0.00001	30%

SMD, standard mean difference; RMT, resting motor threshold; DLPFC, dorsolateral prefrontal cortex; DDLPFC, Bilateral DLPFC; LDLPFC, left DLPFC; RDLPFC, right DLPFC; M1, primary motor cortex.

## Discussion

### Summary of main findings

This network meta-analysis systematically evaluated the difference in efficacy between four stimulation modalities and conventional rehabilitation in improving cognitive dysfunction in patients with PD patients by means of two primary outcomes and two secondary outcomes. A total of 22 RCTs with 1,473 patients were included. The results of the primary outcomes were as follows: HF-rTMS significantly improved MoCA and MMSE scores, and LF-rTMS significantly improved MMSE scores. This suggests that HF-rTMS may show better efficacy in improving cognitive function. Finally, we performed a ranking of the strengths and weaknesses of the intervention modalities, and consistent with the results of the network meta-analysis, HF-rTMS was considered to have better efficacy in both primary outcomes. Among the secondary outcomes, LF-rTMS significantly improved UPDRS I scores and HF-rTMS significantly improved UPDRS II scores. In the MoCA subgroup analyses, it was shown that HF-rTMS significantly improved Visuospatial, Language, Abstraction, and Delayed recall scores, and iTBS significantly improved Language and Delayed recall scores. In conclusion, HF-rTMS has more significant advantages in improving cognitive function in PD patients.

Most of the current research in TMS in PD patients is in the area of confirming effectiveness. A recent meta-analysis (Deng et al., 2022) showed that TMS significantly improves cognitive function in patients with PD patients, and due to methodological limitations inherent in traditional meta-analyses, the authors of the review were only able to perform pairwise comparisons without taking into account the existing evidence network. In addition, to avoid multiple testing, the authors had to combine treatment combinations with different TMS stimulation modalities into a single TMS stimulation group and did not include iTBS, thus potentially masking differences between different TMS modalities. More than 90% of the rTMS included in this study were HF-rTMS, which is somewhat similar to our final conclusion.

In the present study, we further explored the duration of the efficacy of TMS in patients with PD patients. The results showed that the effect of TMS treatment in MoCA scores became insignificant in the first month of follow-up. Whereas in the MMSE scores there was still a significant efficacy at 2 months after the follow-up. The reason for the different effects of the two scales may be that the MoCA includes the assessment of visuospatial and executive functioning in addition to general cognitive functioning, which makes it relatively difficult to improve the MoCA. Through sensitivity analysis, Yang (2023) study was found to be more influential, and after exclusion, the effect of MoCA was lost in the first month, whereas the effect of TMS in MMSE lasted for 1 month, suggesting that the improvement of cognitive functioning in patients with PD patients can last at least 1 month, which is important for guiding the course and period of TMS treatment in clinical practice.

In addition to the stimulation mode, stimulation intensity, stimulation location, and number of stimulation pulses are also important parameters for the application of TMS. A meta-analysis of stimulation parameters when TMS was applied to cognitive function in PD patients was conducted by Jiang Y. et al. (2020). The study concluded that high-frequency TMS stimulation of the DLPFC was able to significantly improve Parkinsonian executive function, but the study did not specifically analyze whether there was a difference in stimulation of the different sides of the DLPFC. In the present study, we grouped the other three factors affecting TMS with the primary outcome metrics. Among the stimulation sites, stimulation of the DDLPFC at 100 intensities showed superior results in terms of MoCA and MMSE, similar to the conclusions of Jiang Y. et al. (2020). No more reliable conclusions were obtained in terms of the number of pulses.

Compared with existing meta-analyses of TMS to improve cognitive function in PD patients, this study refined the classification based on the effectiveness of TMS and explored the comparative efficacy of different TMS modalities on Parkinsonian cognitive function. Moreover, the stimulation parameters were analyzed and compared, providing some insights for future research on TMS parameters. In addition, this paper analyzes and compares the intervention time of TMS, and concludes that TMS has a 1-month improvement effect on cognitive function in PD patients, which provides an important reference significance for the use of TMS treatment cycle in the clinic. It is worth mentioning that in this study, the control group was subdivided into a sham-stimulation group and a rehabilitation training group, and it was found that the treatment effect of the sham-stimulation group was better than that of the conventional rehabilitation group, which may be due to the fact that the sham-stimulation can also produce a sizable placebo effect by inducing the release of dopamine from basal ganglia region, which has already been demonstrated in certain imaging, so the effect of the sham-stimulation should not be neglected in clinical practice (Kim et al., 2008). Based on the results of this study, in the future, clinical research can refine the stimulation mode of TMS around the stimulation parameters to find the optimal stimulation parameter pattern, and can be carried out for the 1-month cycle of treatment to prove whether it is scientifically effective to carry out clinical research. Our network meta-analysis cross-sectional comparison of different TMS stimulation modalities provides valuable new insights into the use of TMS in patients with cognitive impairment in PD patients. We suggest that this analysis be considered as a complement to previous systematic reviews on this topic (Goodwill et al., 2017; Lawrence et al., 2017; Jiang Y. et al., 2020; Deng et al., 2022; He et al., 2022).

### Neurophysiological mechanisms

Current research suggests that altered levels of dopamine (DA) in the midbrain-limbic and midbrain-cortical systems may be associated with non-motor symptoms of PD patients (Kang et al., 2010). The feasibility of TMS for the treatment of PD patients is based on the ability of TMS to induce a rise in intracranial dopamine release, regulate cortical excitability, improve local blood flow in the brain, and regulate the secretion of brain-derived neurotrophic factor (BDNF) in the brain (Helmich et al., 2006; Zhu et al., 2018). Brain-derived neurotrophic factor is a special neurotrophic protein widely expressed in the nervous system. Compared with other parts of the brain, the hippocampus and the cortex contain significantly higher levels of BDNF, and TMS can increase the level of BDNF directly in the brain to improve the cognitive function of the brain. Synapse is a special bridge for nerve cells to connect, TMS can increase intracranial BDNF and change the synaptic plasticity at the same time, and its enhanced connectivity can improve the function of dopaminergic neurons, thus improving the cognitive level of PD patients (Ye and Feng, 2016; Zhao et al., 2019). Acetylcholine (Ach) is an excitatory neurotransmitter that plays an important role in learning and memory, and the occurrence of Parkinson’s is closely related to the reduction of Ach, and the main mechanism lies in the impairment of the cholinergic pathway that leads to the reduction of Ach activity and triggers Parkinson’s (Bonnì et al., 2017; Chi, 2020). Some studies have shown (Zhang et al., 2018) that TMS can increase the activity of acetylcholinesterase and choline acetyltransferase and increase the density of cholinergic neurons, and it is speculated that the mechanism by which TMS improves cognitive and learning functions may be related to the restoration of the activity of the cornu ammonis 1 cholinergic system.

MoCA and MMSE are screening scales for cognitive functions such as attention and memory (Arevalo-Rodriguez et al., 2015; Kang et al., 2018). The frontal lobe is an important regulatory area for cognitive function and is the most common stimulation target for TMS application in patients with cognitive impairment. The results of this study suggest that when the frontal lobe is stimulated, the brain is able to mediate the executive function and attention of the patient, thus improving the patient’s memory, visuospatial processing ability (Burton and Tyson, 2015). HF-rTMS stimulation is able to repeat and regular high-intensity magnetic stimulation of the frontal lobe of the patient’s brain, forming a continuous effect, with specific effects on the frontal cortex, which is more conducive to cognitive improvement than other intervention modes with direct stimulation and increased number of neural connections (Lu et al., 2015). The basal forebrain is an important region of the brain that is rich in cholinergic neurons that release Ach to areas of the brain that are closely related to cognitive processes such as learning, memory, attention, and executive function (Gratwicke et al., 2015). It has been reported (McClelland et al., 2016) that repetitive transcranial magnetic stimulation inhibits activity in other regions of the brain, promotes localized activity, enhances the activity of cholinergic neurons in the basal forebrain, and improves the release of neurotransmitter secretion, such as Ach, thereby promoting the recovery of cognitive function. In addition, stimulation of the DLPFC in the brain can lead to enhanced neural connectivity between the DLPFC and the basal forebrain, prompting changes in the activity patterns of regions such as the basal forebrain, which may include increased activation of cholinergic neurons and enhanced synaptic plasticity of neurons, leading to enhanced release of Ach, and consequently, improved cognitive function (Gratwicke et al., 2015). The frontal lobe was used as the stimulation target in most of the present study, and the high stimulation frequency of HF-rTMS, between 5 and 25, could be an important reason for the more significant effect of HF-rTMS.

UPDRS I and UPDRS II are special assessment scales for evaluating the psychoemotional, behavioral cognitive and daily life ability of PD patients, respectively. TMS treatment can regulate the excitability of the cerebral pallidum and striatal loop, improve the symptoms of cognitive, sleep and motor disorders, enhance the quality of life of the patients, and reduce the negative emotions. Daily living ability is closely related to cognitive function. The ability of daily life includes the participation of several cognitive functions such as executive function, reasoning integration of tasks, etc. TMS can achieve the reconstruction of regional cortical function and the remodeling of brain network system through the cortico-cortical pathway, and promote the effective connection of brain network nerves to enhance the recovery of cognitive function (Hallett et al., 2007). With the improvement of cognitive function, the ability of PD patients in daily life has been improved accordingly.

### Limitations

There are still shortcomings in this study: The majority of the current included literature is a comparison between different intervention models relying on conventional rehabilitation treatment, and lacks two-by-two comparisons between different intervention models, hence the lack of a closed-loop mesh relationship. Due to the limited number of included studies, the subgroup analyses were not categorized by stimulation modality, resulting in potentially biased results. The frequency of high-intensity stimulation spanned from 5 to 25 sessions, making it difficult to determine the frequency of HF-rTMS that is most suitable for patients with PD patients. The duration of intervention varied across studies, and it was not possible to determine the difference between the duration of intervention and efficacy, more high-quality RCTs are still needed in the future to help us increase the sample size and improve the effect estimates.

## Conclusion

The results of this study suggest that high-frequency transcranial magnetic bilateral stimulation of the dorsolateral prefrontal cortex is more effective in improving cognitive deficits in patients with PD patients, and the effect of TMS in improving cognitive function lasts for at least 1 month. Due to the limitations of this study, more high-quality RCTs are still needed in the future to help us increase the sample size and improve the efficacy estimates.

## Data availability statement

The original contributions presented in the study are included in the article/supplementary material, further inquiries can be directed to the corresponding author.

## Author contributions

YY: Conceptualization, Data curation, Formal analysis, Investigation, Methodology, Project administration, Resources, Software, Supervision, Validation, Visualization, Writing – original draft, Writing – review & editing. ZY: Formal analysis, Funding acquisition, Project administration, Validation, Writing – original draft, Writing – review & editing. WC: Conceptualization, Investigation, Software, Writing – original draft. JD: Data curation, Methodology, Writing – original draft. HX: Supervision, Writing – original draft.
